# Comparing coronary artery cross-sectional area among asymptomatic South Asian, White, and Black participants: the MASALA and CARDIA studies

**DOI:** 10.1186/s12872-024-03811-4

**Published:** 2024-03-14

**Authors:** R. T. Short, F. Lin, S. Nair, J. G. Terry, J. J. Carr, N. R. Kandula, D. Lloyd-Jones, A. M. Kanaya

**Affiliations:** 1 University of California San Francisco, San Francisco, USA; 2 Vanderbilt University Medical Center, Nashville, USA; 3 Northwestern University, Evanston, USA

**Keywords:** Coronary artery cross sectional area, South asian, Coronary artery calcium

## Abstract

**Background:**

South Asian individuals have high risk of atherosclerotic cardiovascular disease (ASCVD). Some investigators suggest smaller coronary artery size may be partially responsible.

**Methods:**

We compared the left anterior descending (LAD) artery cross-sectional area (CSA) (lumen and arterial wall) among South Asians in the Mediators of Atherosclerosis in South Asians Living in America (MASALA) study with White and Black participants in the Coronary Artery Risk Development in Young Adults (CARDIA) study, adjusting for BMI, height, and other ASCVD risk factors. We used thin-slice non-contrast cardiac computed tomography to measure LAD CSA. We used linear regression models to determine whether race/ethnicity was associated with LAD CSA after adjusting for demographic factors, BMI, height, coronary artery calcium (CAC), and traditional cardiovascular risk factors.

**Results:**

Our sample included 3,353 participants: 513 self-identified as South Asian (44.4% women), 1286 as Black (59.6% women), and 1554 as White (53.5% women). After adjusting for age, BMI, height, there was no difference in LAD CSA between South Asian men and women compared to White men and women, respectively. After full adjustment for CVD risk factors, LAD CSA values were: South Asian women (19.9 mm^2^, 95% CI [18.8 – 20.9]) and men (22.3 mm^2^, 95% CI [21.4 – 23.2]; White women (20.0 mm^2^, 95% CI [19.4—20.5]) and men (23.6 mm^2^, 95% CI [23.0—24.2]); and Black women (21.6 mm^2^, 95% CI [21.0 – 22.2]) and men (26.0 mm^2^, 95% CI [25.3 – 26.7]). Height, BMI, hypertension, CAC, and age were positively associated with LAD CSA; current and former cigarette use were inversely associated.

**Conclusions:**

South Asian men and women have similar LAD CSA to White men and women, and smaller LAD CSA compared to Black men and women, respectively, after accounting for differences in body size. Future studies should determine whether LAD CSA is associated with future ASCVD events.

**Supplementary Information:**

The online version contains supplementary material available at 10.1186/s12872-024-03811-4.

## Introduction

Numerous studies have demonstrated that people of South Asian background (those with origins from India, Pakistan, Bangladesh, Nepal, Sri Lanka, Bhutan, and the Maldives) experience a higher incidence of atherosclerotic cardiovascular disease (ASCVD) compared to individuals from other racial/ethnic groups [1, 2]. The World Health Organization reported that South Asians account for 60 percent of patients with coronary artery disease globally, while comprising only 23 percent of the world’s population [3]. South Asian populations also tend to develop ASCVD and acute myocardial infarction at younger ages than individuals from other racial/ethnic groups [4], a disparity that persists even after accounting for differences in hypertension, type 2 diabetes, obesity, smoking, and lipid levels [5].

Understanding the multilevel factors that account for higher rates of ASCVD in South Asian populations is important for prevention and treatment. Some experts have suggested that a smaller coronary artery size may be responsible. While a small body of literature comparing coronary artery size between symptomatic South Asians and White individuals exists, these studies were limited by small sample sizes, differences in covariate adjustment, and use of coronary angiography, which primarily measures lumen size [6, 7]. No studies have compared coronary cross-sectional area (including the lumen and arterial wall) across multiple diverse race/ethnic groups among asymptomatic individuals with no clinical evidence of ASCVD.

We utilized two epidemiologic cohorts for the current study: South Asian men and women enrolled in the Mediators of Atherosclerosis in South Asians Living in America (MASALA) and Black and White participants in the Coronary Artery Risk Development in Young Adults study (CARDIA). We compared left anterior descending artery cross-sectional area (LAD CSA) using non-contrast coronary CT images among South Asian, White, and Black participants. We also examined associations of various cardiovascular risk factors on LAD CSA. We hypothesized that South Asian participants would have smaller LAD CSAs than both Black and White participants, and that this association would be attenuated after adjusting for body size (BMI and height) and cardiovascular risk factors.

## Methods

### MASALA study

The MASALA Study is a prospective cohort study that began in 2010–2013 at two clinical sites (University of California San Francisco and Northwestern University). A total of 906 South Asian men and women between ages 40 to 84 years were recruited with community sampling methods (Supplemental Figure 1) [8]. Between 2015–2018, all surviving participants were invited to the second clinical exam [9]. The MASALA protocols were approved by institutional review boards at each site. Signed informed consent was obtained from all participants at every examination.

Of the 749 participants who completed MASALA Exam 2, a total of 701 participants underwent non-contrast CT imaging using cardiac-gated multi-detector CT scans (GE Lightspeed VCT 64 MDCT or Siemens Sensation Cardiac 64 Scanner) and both standard 2.5 mm slices and thin-slice 0.6 mm images were obtained (Supplemental Figure 2). Standard 2.5 mm images were read at the Lundquist Research Institute (Torrance, CA) for total coronary artery calcium (CAC) using published protocols. LAD CSA was measured at Vanderbilt University Medical Center (Nashville, TN) using the thin-slice 0.6 mm images and a dedicated DICOM image processing workstation (Osirix MD, PIXMEO) [10] and an interactive pen computing display (Cintiq, Wacom). This methodology with comparison to coronary CT angiography was recently developed in a study of individuals with HIV [11]. In brief, the isotrophic CT images were displayed in multi-planar reformat mode and the analyst identified the left coronary artery and created a center-line from the origin of the left anterior descending (coronary segment 6) through the mid vessel (American Heart Association, AHA segement 7) [12]. In patients with coronary artery disease, the LAD is the most commonly involved coronary artery and is less prone to motion artifacts [13, 14]. From the centerline, a perpendicular cross-section of the vessel was created and the proximal LAD for a length of 10 mm was identified. The interface between the coronary vessel and the epicardial fat was segemented and the vessel cross-sectional area in mm^2^ was recorded at three consecutive equidistant location perpendicular to the centerline within the 10 mm vessel length in the proximal LAD (segment 6). The main outcome variable was the mean proximal LAD CSA (mm^2^) based on the three measurements. Analysts were blinded to all clinical information and all scans were read in duplicate with low mean difference in values (Pitman’s test of difference in variance *p* < 0.001).

Other covariates assessed at Exam 2 by trained bilingual research staff included participant weight, height, and average seated blood pressure using standardized protocols. Body mass index was calculated as weight (in kg) divided by the square of height (in meters). Hypertension was classified for any participant with an average systolic blood pressure ≥ 140 mmHg or diastolic blood pressure ≥ 90 mmHg, or if the participant was taking an anti-hypertensive medication. Smoking behavior (cigarette use: current, former, never) and alcohol intake (drinks/week) were determined by questionnaires. Fasting blood was drawn to measure fasting plasma glucose, HbA1c, triglycerides, total cholesterol, and high-density lipoprotein (low-density lipoprotein concentration was calculated). A detailed medication inventory was taken by trained staff. Diabetes was classified if fasting glucose was ≥ 126 mg/dL, or 2-h post-challenge glucose ≥ 200 mg/dL, or if the participant was taking a glucose-lowering medication.

### CARDIA study

CARDIA is a prospective cohort study that began in 1985 with 5,115 participants aged 18 to 30 years enrolled at four study sites: Oakland, CA, Birmingham, AL, Chicago, IL, and Minneapolis, MN. The present study includes data from the CARDIA year 25 examination from the 2,840 participants (among 3,499 examined) who underwent cardiac CT. The CARDIA protocols were approved by institutional review boards of the University of Alabama at Birmingham, Kaiser Permanente of Oakland California, University of Minnesota, and Northwestern University. Signed informed consent was obtained from all participants at every examination.

At CARDIA year 25 in 2010–2011, research staff gathered information on age, smoking status, alcohol drinks/week, average seated blood pressure, weight and height measurements, fasting plasma lipids and glucose, and medication use. Participant gender, Black or White race, and years of education were ascertained at the baseline CARDIA examination. BMI was calculated. Hypertension and diabetes were classified using identical clinical, medication inventory, and laboratory criteria as in MASALA.

Participants were invited to undergo non-contrast, ECG-gated multi-detector CT scans at year 25. Scans were performed using multidetector CT systems from General Electric (GE 750HD 64 and GE LightSpeed VCT 64 for Birmingham and Oakland field centers, respectively, GE Healthcare, Waukesha, WI) or Siemens (Sensation 64 for Chicago and Minneapolis field centers, Siemens Medical Solutions, Erlangen, Germany). Standard reconstructions of 35 cm DFOV, 2.5–3 mm slice thickness were used for measurement of CAC as previously described [15, 16] and high-resolution reconstructions at 25 cm DFOV, 0.5–0.6 mm slice thickness and 50 cm DFOV, 0.5–0.6 mm were utilized for measurement of arterial dimensions. CT scans were securely transmitted to CARDIA CT Reading Center at Wake Forest University Medical Center for analyses.

### LAD CSA measurements

CT angiography and non-contrast CT coronary artery calcium calculation are non-invasive methods for characterizing coronary anatomy, stenosis, and plaque burden. In clinical practice, CT coronary angiography uses intravenous administration of iodinated contrast media and high resolution imaging (0.5–0.75 mm slices) and is able to resolve the normal coronary lumen and wall, as well as, atherosclerotic plaque, luminal stenosis and other pathologies. Clinically, non-contrast cardiac CT is used to measure the coronary artery calcium score and more recently the aortic valve calcium score, and uses thicker slices (2.5–3.0 mm slices) without the need for intravascular contrast. We developed methods to measure the size of the coronary arteries using the inherent distinction between the coronary vessel wall and surrounding epicardial adipose tissue by utilizing high resolution, thin slice imaging comparable to CT coronary angiography; however, without administration of intravascular contrast media. This method provides the coronary vessel CSA, a combined measure of the lumen and arterial wall, comparable to adventitial area measured with intravascular ultrasound [17].

In CARDIA, LAD CSA (mm^2^) was measured on 0.5–0.6 mm thick images on a workstation running 3D Slicer (www.slicer.org) with custom plug-in developed in-house. Three experienced analysts identified the proximal LAD and determined the centerline of the vessel as was done in MASALA. On the thin-slice axial images, 3–5 equidistant perpendicular CSA measures were made from the beginning of the proximal LAD. The main outcome variable was the mean proximal LAD CSA (mm^2^) based on 3–5 measures. The intraclass correlation coefficient for proximal LAD CSA in 100 CARDIA scans read by three independent observers was 0.79. The CARDIA and MASALA measurements were performed in the same anatomic segment of the coronary artery, specifically the proximal LAD, segment 6 with the CARDIA protocol allowing a minimum of 3 and maximum of 5 measurements while the MASALA protocol spaced three equidistant and cross-sectional measurements.

We used data from 513 MASALA participants between ages of 42–68 years to have an overlapping age distribution to the CARDIA participants at the year 25 examination. We separated the White and Black self-reported racial groups in CARDIA for comparison to South Asians. Prior CARDIA publications have shown significant differences in body size, cardiovascular risk factors, and CAC burden between Black and White individuals [16]; therefore, we expected there may be differences in LAD CSA between these groups. Our goal was to determine whether adjusting for demographic and cardiovascular risk factors would attenuate any observed differences in LAD CSA among all groups.

### Statistical analysis

We determined the distribution for the LAD CSA and compared frequencies, means, and medians of covariates among the three racial/ethnic groups by gender. We examined the least square means of LAD CSA for each racial/ethnic subgroup by gender across all CAC categories, after adjusting for age, BMI, and height. We purposefully did not adjust for body surface area and weight, as both were highly correlated with BMI and/or height in both cohorts. Next, we examined bivariate associations of traditional cardiovascular risk factors, BMI, height, waist circumference and CAC categories, with LAD CSA. As CAC correlates with coronary plaque burden, we then stratified LAD CSA by CAC category. Next, we created multivariable linear regression models to determine the association between each racial/ethnic group with LDA CSA after adjusting for age and gender, then further adjusted for BMI and height. The final fully adjusted multivariable model also included hypertension, diabetes, current and former cigarette use, LDL-cholesterol, HDL-cholesterol, cholesterol medication use, and CAC categories.

We conducted a sensitivity analysis restricting our sample to participants with no current or former history of smoking, no hypertension, and no detectable CAC. We determined whether LAD CSA varied by race/gender groups after adjusting for age and body size in this subgroup with low burden of risk factors. Because prior studies have adjusted for body size using BSA, we conducted an additional sensitivity analysis using BSA instead of BMI and height. We used SAS 9.4 (Cary, NC) for our statistical analyses.

## Results

We compared key demographic, anthropometric. and cardiovascular risk factors among 513 South Asians participants in the MASALA cohort and 2,840 Black and White participants from CARDIA. Table 1 displays these data stratified by gender.

**Table 1:** Participant characteristics by gender and race/ethnicity, MASALA and CARDIA

Characteristics	**Men**			**Women**		
	South Asian * N* = 285	White * N* = 722	Black * N* = 519	South Asian * N* = 228	White * N* = 832	Black * N* = 767
Age	56.5 ± 6.7	50.6 ± 3.4	49.2 ± 3.8	55.7 ± 6.8	50.7 ± 3.4	49.6 ± 3.8
Educational attainment, yrs	18.5 ± 2.3	15.9 ± 2.6	14.0 ± 2.2	17.8 ± 2.8	16.0 ± 2.4	14.5 ± 2.2
Alcohol drinks/week	2.3 ± 3.9	7.2 ± 10.8	6.3 ± 13.6	0.7 ± 1.9	4.1 ± 6.2	2.2 ± 5.0
Smoking status: Never	202 (70.9%)	454 (63.7%)	293 (57.2%)	218 (95.6%)	477 (58.1%)	471(62.6%)
Past	69 (24.2%)	165 (23.1%)	85 (16.6%)	8 (3.5%)	242 (29.5%)	130 (17.3%)
Current	14 (4.9%)	94 (13.2%)	134 (26.2%)	2 (0.9%)	102 (12.4%)	151 (20.1%)
Height, m	1.71 ± 0.07	1.78 ± 0.07	1.77 ± 0.07	1.58 ± 0.06	1.65 ± 0.06	1.64 ± 0.07
Weight, kg	77.2 ± 11.5	91.7 ± 17.6	93.6 ± 21.0	67.2 ± 11.8	76.6 ± 19.4	88.3 ± 21.7
BMI, kg/m^2^	26.5 ± 3.6	28.9 ± 5.0	29.8 ± 6.0	27.0 ± 4.3	28.2 ± 7.1	32.7 ± 7.6
Waist circumference, cm	96.7 ± 8.9	98.7 ± 13.0	98.0 ± 14.2	91.8 ± 10.8	86.7 ± 15.6	94.7 ± 15.1
Systolic BP, mmHg	128 ± 15	119 ± 14	125 ± 14	123 ± 17	112 ± 15	123 ± 17
Diastolic BP, mmHg	79 ± 9	74 ± 10	78 ± 11	73 ± 9	70 ± 10	78 ± 11
Total cholesterol, mg/dl	179 ± 40	192 ± 37	187 ± 36	199 ± 38	198 ± 34	191 ± 40
HDL-cholesterol, mg/dl	45 ± 12	49 ± 14	53 ± 15	56 ± 15	66 ± 19	61 ± 17
LDL-cholesterol, mg/dl	108 ± 35	115 ± 33	112 ± 34	117 ± 35	111 ± 31	110 ± 34
Triglycerides, mg/dl	141 ± 87	146 ± 127	114 ± 75	129 ± 58	105 ± 66	98 ± 69
Fasting glucose, mg/dl	114 ± 27	103 ± 32	104 ± 33	104 ± 19	93 ± 18	99 ± 30
Hba1c, %	6.2 ± 0.9	5.6 ± 1.0	6.0 ± 1.3	6.1 ± 0.8	5.4 ± 0.6	5.8 ± 1.0
Diabetes	94 (33.0%)	61 (8.4%)	77 (14.8%)	48 (21.1%)	48 (5.8%)	99 (12.9%)
Hypertension	141 (49.5%)	218 (30.2%)	223 (43.0%)	78 (34.2%)	185 (22.2%)	381 (49.7%)
Cholesterol medication use	114 (40.0%)	143 (19.8%)	81 (15.7%)	52 (22.8%)	86 (10.4%)	128 (16.7%)
Diabetes medication use	71 (24.9%)	36 (5.0%)	53 (10.2%)	32 (14.0%)	25 (3.0%)	73 (9.5%)
Hypertension medication use	100 (35.1%)	168 (23.3%)	172 (33.1%)	59 (25.9%)	141 (16.9%)	307 (40.0%)
CAC score 0	87 (30.5%)	384 (53.2%)	348 (67.1%)	159 (69.7%)	693 (83.3%)	625 (81.5%)
CAC score 1–99	107 (37.5%)	218 (30.2%)	109 (21.0%)	47 (20.6%)	102 (12.3%)	99 (12.9%)
CAC score ≥ 100	91 (31.9%)	120 (16.6%)	62 (12.0%)	22 ( 9.6%)	37 ( 4.4%)	43 ( 5.6%)

South Asian participants were, on average, approximately 6 years older and had 2–3 more years of education than White and Black participants. Lifetime smoking was lower among South Asians; notably, 71% and 96% of South Asian men and women participants, respectively had never smoked. Black women had the highest prevalence of hypertension (49.5%). South Asian men had the highest use of cholesterol-lowering medication use (40%) of any group. Anti-hypertensive medication use varied among groups, with Black women having the highest use (40%) and White women with the lowest (17%). South Asian men and women had lower anthropometric measures (weight, height, BMI, and waist circumference) than Black and White participants in CARDIA. After adjusting for age, South Asian men had the highest prevalence of any CAC (58% in South Asian men, 48% in White men, and 38% in Black men). South Asian and Black women had similar prevalences of CAC (approximately 20%) higher than among White women (17%).

**Fig. 1:**
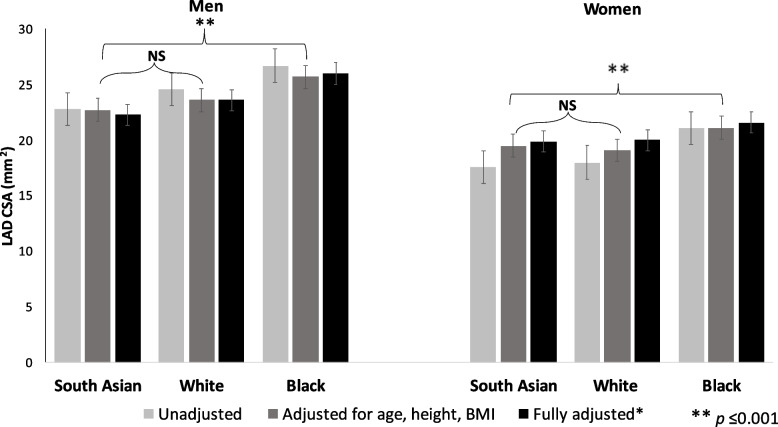
Left anterior descending cross-sectional area (lumen and arterial wall, in mm^2^) with sequential adjustment for body size and cardiovascular risk factors; MASALA and CARDIA studies. *Adjusted for age, BMI, height, hypertension, smoking, diabetes, HDL-cholesterol, LDL-cholesterol, cholesterol medication use, and CAC categories

In unadjusted analyses, South Asian men and women had smaller average LAD CSA than both Black and White participants in CARDIA (Fig. 1).

We used BMI and height as distinct measures of body size (*r* < 0.20), and did not adjust for weight, waist circumference, or body surface area as they were highly correlated with BMI and height in both cohorts. There was no significant difference in LAD CSA between South Asian men and women compared to White men and women after adjusting for age and body size. Further adjustment for ASCVD risk factors showed that South Asian men had somewhat smaller LAD CSA compared to White men (22.3 mm^2^ (99% CI 21.4 – 23.2) vs. 23.6 mm^2^ (99% CI 23.0 – 24.2), *p* = 0.009), but there remained no significant difference between South Asian and White women. Adjustment for body size and other risk factors did not substantially attenuate the differences in LAD CSA between South Asian and Black men and women.

**Fig. 2:**
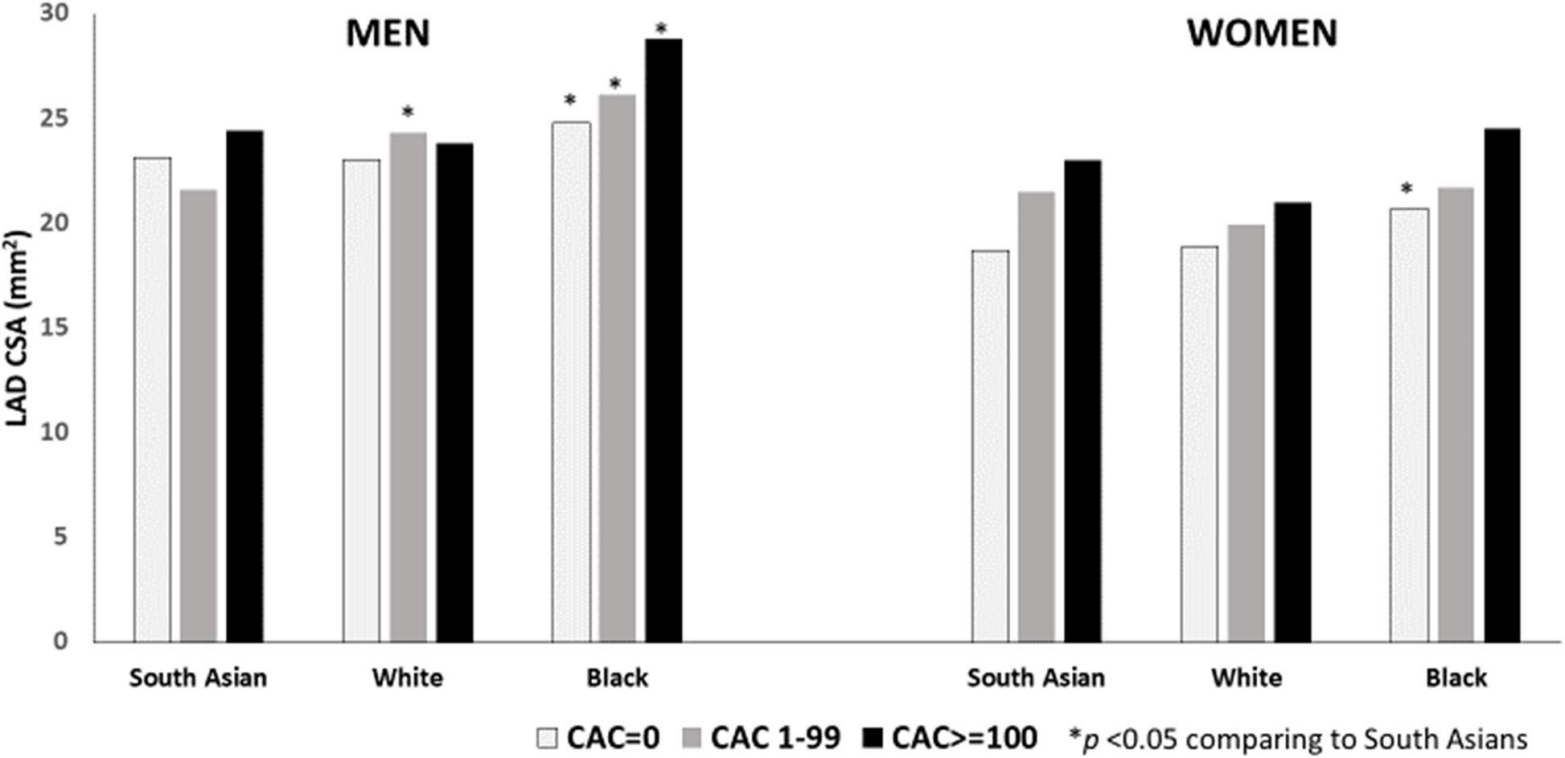
Left anterior descending cross-sectional area (lumen and arterial wall, in mm^2^) by coronary artery calcium score by gender and racial/ethnic group adjusted for age, BMI and height; MASALA and CARDIA studies

We found a positive, graded association between greater CAC burden and LAD CSA for both men and women and across all three racial/ethnic groups after adjusting for age, BMI, and height (Fig. 2).

Across all three racial/ethnic subgroups, CAC scores of 1–99 were associated with a 1.0 mm^2^ higher LAD CSA, and CAC scores of 100 or greater were associated with 2.3 mm^2^ higher LAD CSA compared to those with CAC of zero after adjusting for age, gender, BMI and height.

In multivariable models adjusted for age, gender, BMI, and height we found that South Asian participants had smaller LAD CSA than Black participants, but there was no difference between South Asian and White participants (Table 2).

**Table 2:** Associations of race/ethnicity and cardiovascular risk factors with LAD CSA, MASALA and CARDIA studies

**LAD CSA Beta Coefficient, Standard Error, *p*-value**			
	Adjustment for age and gender *N* = 3353	Adjustment for age, gender, BMI, height *N* = 3352	Fully adjusted^a^ *N* = 3269
South Asian participants	0 (reference)	0 (reference)	0 (reference)
White participants	**1.86 (0.39), < 0.001**	0.27 (0.39), 0.49	0.78 (0.41), 0.05
Black participants	**4.69 (0.42), < 0.001**	**2.28 (0.42), < 0.001**	**2.75 (0.44), < 0.001**
Male gender	**6.05 (0.25), < 0.001**	**4.35 (0.32), < 0.001**	**3.76 (0.34), < 0.001**
Age, year	**0.13 (0.03), < 0.001**	**0.14 (0.03), < 0.001**	**0.08 (0.03), 0.006**
Height, cm	-	**15.1 (1.73), < 0.001**	**15.2 (1.73), < 0.001**
BMI, kg/m^2^	-	**0.32 (0.02), < 0.001**	**0.28 (0.02), < 0.001**
HTN	-	-	**1.21 (0.27), < 0.001**
Diabetes	-	-	0.16 (0.39), 0.68
Current smoker	-	-	**-1.12 (0.34), 0.001**
Former smoker	-	-	**-0.77 (0.29), 0.01**
Never smoker	-	-	0 (reference)
HDL-cholesterol, mg/dL	-	-	-0.01 (0.01), 0.42
LDL-cholesterol, mg/dL	-	-	0.00 (0.00), 0.51
Cholesterol med use	-	-	-0.15 (0.34), 0.65
CAC score 0	-	-	0 (reference)
CAC score 1–99	-	-	**1.01 (0.31), 0.001**
CAC score ≥ 100	-	-	**2.47 (0.42), < 0.001**

^a^Adjusted for gender, age, BMI, height, hypertension, smoking, diabetes, HDL-cholesterol, LDL-cholesterol, cholesterol medication use, and CAC categories

On average, LAD CSAs were 2.3 mm^2^ larger for Black individuals compared to South Asian participants. Adjusting for body size, cardiovascular risk factors, and CAC amplified the difference between LAD CSA among racial/ethnic groups, with greater LAD CSA among Black (2.7 mm^2^) and White participants (0.8 mm^2^) compared to South Asian individuals. Height, male gender, hypertension, CAC 1–99, CAC ≥ 100, BMI, and age were all associated with higher LAD CSA, whereas current and former smoking were inversely associated.

In a sensitivity analysis restricted to 1,401 participants (61 South Asian men, 119 South Asian women, 279 White men, 496 White women, 161 Black men, and 285 Black women) with no history of smoking, hypertension, or detectable CAC, we found no significant differences in LAD CSA comparing South Asian and White men and women, respectively, after adjusting for age, BMI and height. Black men and women had greater LAD CSA compared to South Asian men and women (Supplemental Figure 3).

In another sensitivity analysis using BSA instead of BMI and height as a surrogate for body size yielded similar results (Supplemental Table, Supplemental Figure 4). In models using BSA for body size adjustment, South Asian participants had significantly smaller LAD CSAs compared to Black participants while there was no significant difference between South Asians and White participants in both minimally and fully adjusted models (Supplemental Table).

## Discussion

South Asian men and women have smaller left anterior descending coronary artery cross-sectional area compared to other groups, but the differences between South Asian and White individuals were attenuated by adjustment for age and body size. Black men and women had greater LAD CSA than both South Asian and White men and women, respectively, even after accounting for differences in body size and cardiovascular risk factors. This study includes participants without clinical coronary heart disease from two large community-based cohorts, extending what has been previously reported in symptomatic clinical populations.

We identified seven observational studies from 1995 to 2017 comparing LAD diameters between South Asian and White patients [6, 18–23]. Five of these studies found that South Asians had smaller diameters after adjusting for body surface area [6, 18, 20, 21, 23]; two studies found no difference between groups, which is consistent with the current study [19, 22]. Regardless, these existing studies have similar limitations: small sample sizes, inclusion of only symptomatic patients, and requirement of invasive coronary angiography. Additionally, although these studies adjusted coronary measurements for body size, they did not adjust for cardiovascular risk factors. Therefore, existing knowledge of this topic is limited. Our study is novel in that it (1) utilized non-invasive methods for coronary artery size characterization, (2) considered the associations of subclinical coronary artery disease as measured by CAC and other traditional risk factors, and (3) directly compared South Asians to both Black and White participants from two large, well characterized epidemiologic cohorts.

Coronary artery calcium score is a strong independent predictor of ASCVD [24, 25]. Multiple studies have demonstrated the direct association between CAC burden and cardiovascular disease, fatal and non-fatal myocardial infarction, and mortality [26–30]. As expected, we observed that individuals with higher CAC had larger LAD CSA, regardless of gender or race/ethnic background. Additionally, LAD CSA was similar in all groups of women across CAC categories, but differed primarily between South Asian and Black men. However, adjusting for CAC level did not explain the smaller coronary size seen in South Asian participants compared to Black individuals. Furthermore, as both South Asian and Black populations have been shown to have higher incidence of ASCVD compared to White populations, differences in LAD CSA and CAC will not explain these disparities in ASCVD. Makaryus et al. have posited that higher CAD burden among South Asians may be due to the lesser atheroma mass required to create unstable plaques [23]. Nonetheless, this relationship between CAC and coronary size is consistent with the findings of Glagov et al. in 1987, who showed that coronary arteries with larger plaque burden tend to have larger diameter, owing to compensatory “positive” or “outward” remodeling such that luminal area is initially preserved despite plaque growth [31]. Our findings suggest this mechanism is preserved across race/ethnicity and gender. Few studies have examined the relationship between CAC score and coronary cross-sectional area. In 2014, Hamirani et al. used coronary CT data from 140 symptomatic patients to compare the associations of CAC, gender, smoking, BMI, and hypertension with left main and right coronary artery diameters. They also found a direct relationship between CAC and vessel diameter [32]. In 2022, using the same methods as the current study, Werede et al. observed that, in persons with HIV, larger LAD CSA was associated with a lower CD4 T-cell count and lower circulating interleukin-10 levels [14]. Our study extends these results to community-based samples among participants from three different racial/ethnic groups.

We observed an unexpected association between current smoking and lower LAD CSA in the fully adjusted model. Smoking is been associated with greater coronary atherosclerosis; CAC score and incidence of ASCVD events are higher among individuals who smoke [33]. In accordance with our findings that CAC score and coronary area were directly proportional, we expected smoking to be associated with larger coronary size. Interestingly, in a recent review, Salehi et al. reinforced that smoking was associated with greater severity of atherosclerosis, but found that location of plaques and number of coronary arteries involved were not reliably associated with smoking [34]. Nicotine is known to expedite atherosclerosis, impair coronary vasodilation and promote vasoconstriction at the level of the endothelium [35, 36]; perhaps these endothelial changes outweigh the natural compensatory mechanism for stenosed coronaries to dilate, as described by Glagov et al. Further, Quillen et al. describe temporary vasoconstriction within thirty minutes of smoking, which may have impacted our results [37]. Additional research is needed to further elucidate these relationships.

A potential limitation of the current study is that it draws samples from two different study cohorts: MASALA and CARDIA. Although both cohorts included asymptomatic community-dwelling participants and used similar cardiac CT protocols and reading center for LAD CSA measurements, differences in samples may exist, such as temporal, geographic, and CT acquisition factors. Our study’s cross-sectional design limits causal inference. Additionally, while our analysis focuses on the LAD artery, it is possible that differences exist in atherosclerosis distribution among racial/ethnic groups. Finally, self-reported race is a social, not a biological construct, though we operationalized South Asian ethnicity as a distinct ancestral group from White and Black race for this analysis. Our analysis did not account for measures of socioeconomic status, birth and current home environment, geographic location in the U.S., and other structural factors which may be associated with self-identified race/ethnicity and may also affect coronary size.

## Conclusion

South Asian men and women have smaller coronary artery cross-sectional area than White and Black individuals, but the differences between South Asian and White individuals may be explained by differences in body size. CAC burden, a correlate of overall plaque burden, was associated with larger LAD CSA among all groups, but plaque burden did not account for the smaller coronary size seen in South Asian participants. These results represent a novel finding in our attempt to understand South Asian risk for cardiovascular disease. Future studies following these cohorts should determine whether LAD CSA is associated with cardiovascular event incidence, and whether this may help explain the higher ASCVD risk among South Asians.

### Supplementary Information

**Supplementary Material 1. **

**Supplementary Material 2. **

## Data Availability

CARDIA study: The datasets generated and/or analyzed during the CARDIA study are available from the US National heart, Lung and Blood Institute for Years 0–30. Information on how to request this data can be found at https://www.cardia.dopm.uab.edu/study-information/nhlbi-data-repository-data/use-of-nhlbi-data-repository-datasets. MASALA study: The datasets used and/or analyzed during the MASALA study available from the corresponding author on reasonable request: https://www.masalastudy.org/for-researchers.

## Abbreviations

MASALA: Mediators of Atherosclerosis in South Asians Living in America Study
CARDIA: Coronary Artery Risk Development in Young Adults Study
LAD: Left anterior descending coronary artery
CSA: Cross sectional area
CAC: Coronary artery calcium score
CAD: Coronary artery disease
ASCVD: Atherosclerotic cardiovascular disease
CVD: Cardiovascular disease
