# Supplementary material for: Comparing coronary artery cross-sectional area among asymptomatic South Asian, White, and Black participants: the MASALA and CARDIA studies
Source: BMC Cardiovasc Disord. 2024 Mar 14;24:158. doi: 10.1186/s12872-024-03811-4 (PMC10938784; doi:10.1186/s12872-024-03811-4)
Supplement: Supplementary file 1 — Supplementary Material 1. [file 12872_2024_3811_MOESM1_ESM.docx]

Supplemental Figure 1. MASALA and CARDIA cohort sizes at inception and time of current study


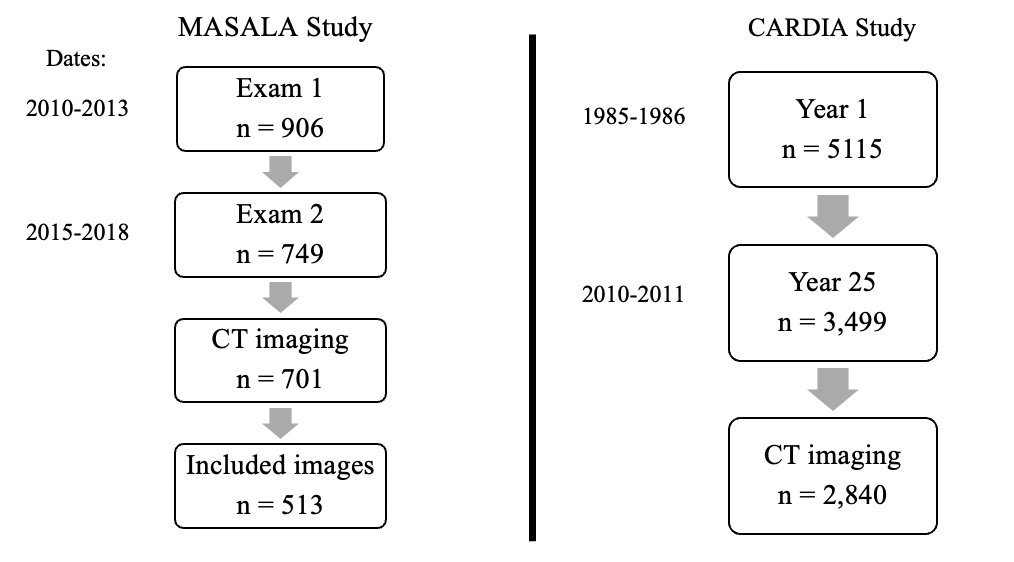


Supplemental Figure 2. MASALA and CARDIA CT evaluation methodology


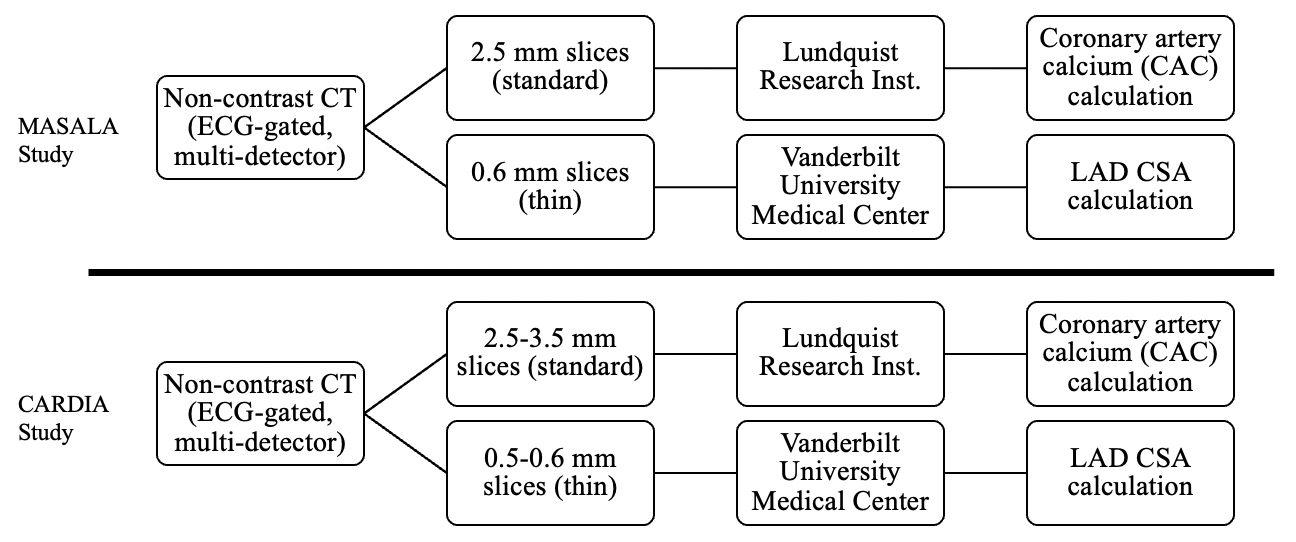


Supplemental Figure 3: Left anterior descending cross-sectional area comparisons among participants with no history of smoking and hypertension, and no detectable CAC; MASALA and CARDIA studies

Supplemental Figure 4. Left anterior descending cross-sectional area with sequential adjustment for body surface area (BSA) and cardiovascular risk factors; MASALA and CARDIA studies


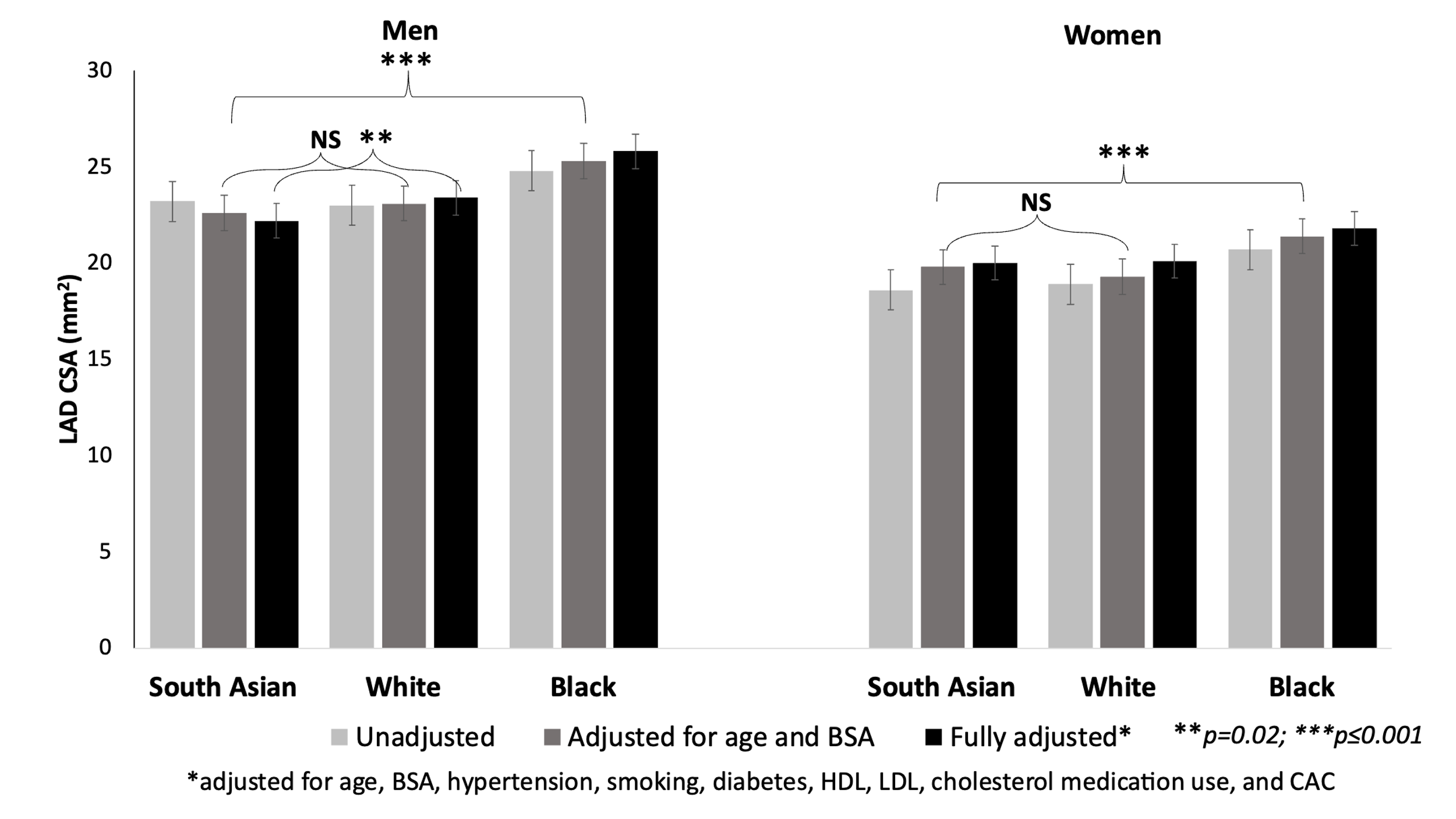


Supplemental Table: Associations of race/ethnicity, BSA, and cardiovascular risk factors with LAD CSA, MASALA and CARDIA studies

| **LAD CSA Beta Coefficient, Standard Error, p-value** | | | |  |
| --- | --- | --- | --- | --- |
|  | Adjustment for age and gender N=3353 | Adjustment for age, gender, BSA  N=3351 | Fully adjusted*  N=3269 | |
| South Asian participants | 0 (reference) | 0 (reference) | 0 (reference) | |
| White participants | **1.86 (0.39),** **<0.001** | 0.09 (0.38), 0.82 | 0.73 (0.40), 0.07 | |
| Black participants | **4.69 (0.42), <0.001** | **2.23 (0.42), <0.001** | **2.74 (0.44), <0.001** | |
| Male gender | **6.05 (0.25), <0.001** | **3.70 (0.26), <0.001** | **3.39 (0.28), <0.001** | |
| Age, year | **0.13 (0.03), <0.001** | **0.15 (0.03), <0.001** | **0.08 (0.03), 0.005** | |
| BSA, (unit) | - | **10.8 (0.56), <0.001** | **9.65 (0.60), <0.001** | |
| HTN | - | - | **1.27 (0.27), <0.001** | |
| Diabetes | - | - | 0.20 (0.39), 0.61 | |
| Current smoker | - | - | **-1.12 (0.34), 0.001** | |
| Former smoker | - | - | -0.75 (0.29), 0.01 | |
| Never smoker | - | - | 0 (reference) | |
| HDL-cholesterol, mg/dL | - | - | -0.01 (0.01), 0.40 | |
| LDL-cholesterol, mg/dL | - | - | 0.00 (0.65), 0.52 | |
| Cholesterol med use | - | - | -0.15 (0.34), 0.65 | |
| CAC score 0 | - | - | 0 (reference) | |
| CAC score 1-99 | - | - | **1.02 (0.31), 0.001** | |
| CAC score ≥100 | - | - | **2.48 (0.42), <0.001** | |
|  |  |  |  | |
