# Supplementary material for: Comparing coronary artery cross-sectional area among asymptomatic South Asian, White, and Black participants: the MASALA and CARDIA studies
Source: BMC Cardiovasc Disord. 2024 Mar 14;24:158. doi: 10.1186/s12872-024-03811-4 (PMC10938784; doi:10.1186/s12872-024-03811-4)
Supplement: Supplementary file 2 — Supplementary Material 2. [file 12872_2024_3811_MOESM2_ESM.pdf]

UNIVERSITY OF CALIFORNIA, SAN FRANCISCO
CONSENT TO PARTICIPATE IN A RESEARCH STUDY

Study Title: Mediators of Atherosclerosis in South Asians Living in America (MASALA study)

This is a research study to learn what factors lead to heart disease and stroke among South Asians. The study researchers, Dr. Alka Kanaya, and her associates from the UCSF Department of Medicine will explain this study to you.

Medical research studies include only people who choose to take part. Take your time to make your decision about participating. You may discuss your decision with your family and friends and with your health care team. If you have any questions, you may ask your study doctor.

You are being asked to take part in this study because you are a current participant in the MASALA Study.

Why is this study being done?

The purpose of this study is to measure the progression of cardiovascular disease in South Asians and compare these progression rates to those in other U.S. ethnic groups. The National Institutes of Health (NIH) is providing funding for this study.

How many people will take part in this study?

About 1000 men and women will participate in this study at two clinical sites. At UCSF, up to 550 people will participate in this study.

What will happen if I take part in this research study?

Clinic Visit
The study will require a clinical visit arranged at the Clinical Research Center (CRC) at San Francisco General Hospital or at the Moffitt/Long Hospital at UCSF. For this visit, we ask that you drink at least 1 liter of water the night before the study visit and at least 1 liter the morning of the study visit. You should not eat or drink anything other than water for at least 12 hours before you report to the study site.

- The study will be discussed with you, and you will be asked to review and sign the consent form.

If you agree to be in this study, the following will happen:

- *Urine sample:* We will check your urine for the presence of small amounts of protein. If you are a woman who has not gone through menopause, we will check a urine pregnancy test. If you are pregnant, you would not undergo the cardiac CT scan.

VOID

- *Blood samples:* Blood samples will be collected from an arm vein for measurement of routine laboratory chemistries, blood fats, and hormone levels. The total amount of blood that will be taken for these tests is approximately four tablespoons. Two tubes of your blood will be sent to a commercial laboratory for routine laboratory tests like glucose, cholesterol, kidney and liver tests. We will share these results with you, and you may wish to discuss these results with your own doctor. The remainder of these blood samples will be stored temporarily to run in batches to be used for research purposes only and will not be sold. All information collected during your clinic exams is private and all blood samples will be stored anonymously will all personal identifiers removed. Thus, you will not be contacted with the results of any testing done on these stored samples.

- *2-hour Oral Glucose Tolerance Test (OGTT):* You will have a special type of glucose tolerance test called OGTT to find out how your body uses sugar and insulin. Insulin is a hormone that your body makes to help it use sugar. During the test you will be resting. You will be asked to drink a sugar solution, with 75 grams of glucose. After 2-hours, a blood sample will be drawn from a vein in your arm. The total amount of blood taken for this test is about 1 teaspoon at each time-point. You will not be allowed to eat or drink anything except water, or to smoke cigarettes, until this test is completed. You will receive a meal after the final blood sample is drawn. If you are taking medications for diabetes already, we will not conduct this test on you.

- *Questionnaires:* We will give you questionnaires to fill out which will include information on your health-related behaviors such as smoking, drinking alcohol, general health, and information about your spiritual beliefs. The questionnaire should take less than 30 minutes to complete. A few questionnaires may be mailed to you for completion prior to the visit to shorten the time of the visit.

    - If you have not completed your MASALA Social Network Visit:
        - A research staff will administer the questionnaires to you which will include information on your health-related behaviors such as medication use, physical activity, and diet.
        - We will also be asking you detailed questions about your social connections and networks, the health behaviors of your social contacts, and how the social network is influencing your health.

        These questionnaires should take about two hours to complete.

- *Height, weight, waist and hip circumference and blood pressure:* You may need to change into a hospital gown and pajama bottoms for these measurements. We will share these results with you, and you may wish to discuss these results with your own doctor.

This clinical visit will take about 2-3 hours.

- *Cardiac CT scan:* After completing the procedures described above at the SFGH CRC, we will arrange for you to go to the Radiology Department at UCSF for a CT scan on your heart. For these scan, you will lie on your back with both arms stretched above your head. We will

VOID

take X-rays of your heart. This procedure takes approximately 10 minutes. We will give you the results of the coronary artery calcium score from the cardiac CT test so that you may discuss these results with your doctor. However, since we will be batching these tests together for many study participants, the test result may take several months to receive.

This CT scan appointment will take approximately 30 minutes.

Annual Follow-up Telephone Call

We will contact you by phone, email, or regular mail about annually after your Clinic Visit and ask you about your health since the last contact. If you are unable to answer questions yourself, we may contact a person you have named (a family member or close friend) who could answer questions for you. If you are hospitalized or admitted to a convalescent or nursing home, we will ask that institution for your records. We will review the records to determine the reason for your admission and your diagnosis. We may request records from your doctor for certain office or clinic visits to determine if you have been diagnosed with one of the diseases that MASALA is studying. We may also request Medicare records. We would also want to access information about the cause of death, if anything unfortunate should happen during the study. We may request death certificates or coroner's reports from the Department of Health. If you should lose contact with the MASALA study, we request your permission to contact you or your relatives or friends. We also request your permission to use a commercial locator service to find your current address and telephone number.

Optional collection of blood samples for future research:

We would like your permission to collect and store extra blood for future research. We would like your permission to collect two extra tubes of blood for Genetic Testing. This is optional.

GENETIC TESTING: In the future, there may be discoveries about other important things to measure in DNA and mRNA in relation to cardiovascular disease. Therefore, we would like your permission to save some of your blood for DNA and mRNA measurements for up to 25 years for future research. There will be no charge, and you will not receive any payment or financial benefit from any findings. You will not be told the results of this extra research. Participation in this extra research is voluntary. Please let us know whether you are willing to allow this extra research by initialing the appropriate line at the end of this form.

- Confidentiality of Gene Testing Results
  Your blood samples will be stored at a research laboratory at the UCSF. The information will be analyzed under code so identification of the individual linked to the blood sample can only be identified by obtaining permission from the study investigators.

- Consequences of DNA typing
  We will be genotyping specific genetic sequences or polymorphisms that are related to cardiovascular disease and risk factors. The results of these genetic studies will remain confidential and will not be released to you.

VOID

- Access to Genetic Information
  You will not be told the results of the genetic tests that will be performed on your blood. However, if the investigators discover clinically important information from this study that may alter our current understanding of cardiovascular disease then they will publish this finding so that the information is available to your doctor.

- Secondary Use of the Genetic Sample
  It is possible that identification of genes related to cardiovascular disease that could lead to the development of new screening or diagnostic tests or new treatments for these and related disorders.

What will happen if I agree to donate my blood for GENETIC TESTING?

If you agree to let researchers collect and store your specimens for future research, the following will happen:

- If you give us permission to save some blood for this future measurement, we will collect extra blood, and instead of discarding your leftover specimens we will save them in what is called a "tissue bank" for possible future research:

    - GENETIC TESTING: we will collect an extra tablespoon of blood

- The samples will be coded by ID number only, not by names, and will be accessed only by our research team.
- We also will collect and save information from your research medical record, including things like your age, ethnicity, blood pressure, height, weight, waist/hip measurements, coronary calcium (per the CT scan), results of other blood tests, information from questionnaires that you complete including your general health and personal history.
- We do not know for sure if your specimens or information from your research medical record will be used, but they might be used in research to learn more about risk factors associated with the cardiovascular disease and/or may result in new tests or discoveries.
- We may give your specimens and certain medical information about you (for example, diagnosis, blood pressure, age) to other scientists or companies not at UCSF but we will not give them your name, address, phone number, or any other information that would identify you. Reports about any research will not be given to you or your doctor. Sometimes specimens are used for genetic research (about diseases that are passed on in families). Even if we use the specimen for genetic research, we will not put the results in your medical record. The research will not change the care you receive. Your specimen and any information about you will be kept until it is used up or destroyed. It may be used to develop new drugs, tests, treatments or products. In some instances these may have potential commercial value. Your personal health information cannot be used for additional research without additional approval from either you or a review committee.
- Your specimens will be kept for up to 25 years. If you decide later that you do not want your specimens and information to be used for future research, you can tell us, and we will destroy any remaining identifiable specimens and information if they are no longer needed for your

VOID

care. However, if any research has already been done using portions of your specimens, the data will be kept and analyzed as part of those research studies.

How long will I be in the study?

Participation in the study will take a total of about 3 to 5 hours for the clinical visit and CT scan.

We will be calling you by phone every year after this visit to see if you have had any new medical conditions since your clinical visit. If you have had any cardiovascular disease problems arise, we ask your permission to contact your physician(s) to obtain your medical records to verify the condition for our study records.

If you should move and/or change jobs and lose contact with the MASALA study, we request your permission to contact you or your relatives or friends whose names and addresses you have provided and/or to utilize a commercial locator service to find your current address and telephone number.

Can I stop being in the study?

Yes. You can decide to stop at any time. Tell the study staff if you are thinking about stopping or decide to stop. S/he will tell you how to stop your participation. The study doctor may stop you from taking part in this study at any time if she believes it is in your best interest, if you do not follow the study rules, or if the study is stopped.

What side effects or risks can I expect from being in the study?

- *Radiation Exposure:* The amount of radiation you will be exposed to is relatively small. These doses of radiation could be potentially harmful, but the risks are so small that they are difficult to measure. If you have had a lot of x-rays already or if you might be pregnant, you should discuss this with the investigator. If your urine pregnancy test is positive, you will not undergo the cardiac CT scan.

- *Cardiac CT scan:* CT scans involve the risks of radiation (see above). We will not be administering any contrast agent. Having a CT scan may mean some added discomfort for you. In particular, you may be bothered by feelings of claustrophobia when placed inside the CT scanner. Participants who have arthritis may experience some discomfort lying still on their back for 10 minutes, but positioning aids and cushions will be used to minimize this discomfort. We will be providing you with results of the calcium score of your heart arteries. We will encourage you to seek care from your own doctor for further evaluation or treatment in the event that we find significant hardening of these arteries. The knowledge of this result may cause you some anxiety. If you do not have a doctor, we will provide you with a list of county health departments where you can inquire about local physician services based upon your health insurance coverage.

- *Oral glucose tolerance test:* Some people feel nauseated after drinking the glucose for the test, and may also vomit. There is less than 1% risk of a drop in blood glucose level towards the end of this test. We will monitor you closely for any symptoms of low blood sugar (such as weakness, hunger, sweating, and feeling nervous or restless). The study will be discontinued if any of these low glucose symptoms occur.
- *Blood Draw (Venipuncture):* The risks of drawing blood include temporary discomfort from the needle stick, bruising, and, rarely, infection. The total amount of blood drawn during your visit at the CRC will not exceed 5 tablespoons.

- *Genetic Testing:* There is a risk someone could get access to the personal information in your medical records or other information researchers have kept about you. Someone might be able to trace this information back to you. The researchers believe the chance that someone will identify you is very small, but the risk may change in the future as people come up with new ways of tracing information. In some cases, this information could be used to make it harder for you to get or keep a job. There are laws against misuse of genetic information, but they may not give full protection. The researchers believe the chance these things will happen is very small, but cannot promise that they will not occur.

- *Confidentiality:* Participation in research may involve a loss of privacy, but information about you will be handled as confidentially as possible. Your name will not be used in any published reports about this study.

- *Unknown Risks*: The experimental treatments may have side effects that no one knows about yet. The researchers will let you know if they learn anything that might make you change your mind about participating in the study.

For more information about risks and side effects, ask your study doctor.

Are there benefits to taking part in the study?

There is no direct benefit to you from participating in this study. You will receive the results of several of your blood tests, physical examination findings, and heart CT scan. However, we hope that the information gained from the study will help the researchers to understand risk factors for cardiovascular disease among South Asians and to compare these risks with other ethnic groups in the United States.

What other choices do I have if I do not take part in this study?

You may elect not to participate in this study.

How will information about me be kept confidential?

We will do our best to make sure that the personal information in your medical record is kept private. However, we cannot guarantee total privacy. Your personal information may be given

VOID

out if required by law. If information from this study is published or presented at scientific meetings, your name, social security number, and other personal information will not be used. All data collected in this study will be encrypted (electronically scrambled) and sent to the Data Coordinating Center at the UCSF, where it will be stored in a secure database. Study records may be kept indefinitely for analysis and follow-up.
Organizations that may look at and/or copy your medical records for research, quality assurance, and data analysis include:

- UCSF's Committee on Human Research
- The National Institute of Health which is involved in keeping research safe for people.

What are the costs of taking part in this study?

You will not be charged for any of the study activities.

Will I be paid for taking part in this study?

In return for your time and effort associated with participation in this study, you will be reimbursed with a $25 VISA gift card or $25 cash for the completion of the clinical visit. If you completed the MASALA Social Network Questionnaires at this visit, you will receive an additional $25 VISA gift card or $25 cash. If we need to schedule your CT scan on a different day from your main clinical visit, you will receive an additional $25 VISA gift card or cash for your time.

<u>Travel and Transportation Reimbursement:</u>

If your visit is at Zuckerberg San Francisco General Hospital, you will be reimbursed you for your transportation expenses to and from the clinic visit. If your visit is at UCSF Parnassus Hospital, you will receive parking stickers for your parking costs at Millberry Union Parking Garage (500 Parnassus Avenue) or be reimbursed for your transportation expenses to and from the clinic visit. You will be paid by cash or check for your transportation expenses. If your travel expenses exceed $150, you should receive a check four to six weeks after your last visit. You must give the researchers your address and Social Security number so the check can be processed.

If you have moved from the San Francisco Bay Area and are traveling to attend the Study Visit, you will be paid $250 by check, and you should receive the check four to six weeks after your last visit. You must give the researchers your address and Social Security number so the check can be processed.

What happens if I am injured because I took part in this study?

It is important that you tell your study doctor, Alka Kanaya, MD, if you feel that you have been injured because of taking part in this study. You can tell the doctor in person or call her at (415) 353-7919.

VOID

Treatment and Compensation for Injury: If you are injured as a result of being in this study, the University of California will provide necessary medical treatment. The costs of the treatment may be billed to you or your insurer just like any other medical costs, or covered by the University of California or the study sponsor, National Institutes of Health, depending on a number of factors. The University and the study sponsor do not normally provide any other form of compensation for injury. For further information about this, you may call the office of the Committee on Human Research at 415- 476-1814.

What are my rights if I take part in this study?

Taking part in this study is your choice. You may choose either to take part or not to take part in the study. If you decide to take part in this study, you may leave the study at any time. No matter what decision you make, there will be no penalty to you and you will not lose any of your regular benefits. Leaving the study will not affect your medical care. You can still get your medical care from our institution.

We will tell you about new information or changes in the study that may affect your health or your willingness to continue in the study.

In the case of injury resulting from this study, you do not lose any of your legal rights to seek payment by signing this form.

Who can answer my questions about the study?

You can talk to the study doctor about any questions, concerns, or complaints you have about this study. Contact the study doctor, Alka Kanaya, MD at (415) 353-7919. If you wish to ask questions about the study or your rights as a research participant to someone other than the researchers or if you wish to voice any problems or concerns you may have about the study, please call the Office of the Committee on Human Research at 415-476-1814.

VOID

Check List for Optional Items:

1. Genetic Testing:

- ☐ Yes, I consent to allowing the researchers make DNA and mRNA from my blood for genetic testing.
- ☐ No, I do not wish the researchers make or study my DNA and mRNA.

2. Future contact with this study:

- ☐ This consent form only covers study assessments up to 4 years. If we are able to follow up with participants in this study in future years, we will contact you if you check this box.

3. Future contact for other studies:

- ☐ There are other studies that you may be eligible for in the future. UCSF may contact you to ask you to participate in other research studies if you check this box.

CONSENT

You have been given copies of this consent form and the Experimental Subject's Bill of Rights to keep.

You will be asked to sign a separate form authorizing access, use, creation, or disclosure of health information about you.

PARTICIPATION IN RESEARCH IS VOLUNTARY. You have the right to decline to participate or to withdraw at any point in this study without penalty or loss of benefits to which you are otherwise entitled.

If you wish to participate in this study, you should sign below.

VOID

__________ ______________________________
Date Participant's Signature for Consent

__________ ______________________________
Date Person Obtaining Consent
